# Characterization of Collagen Fibers (I, III, IV) and Elastin of Normal and Neoplastic Canine Prostatic Tissues

**DOI:** 10.3390/vetsci6010022

**Published:** 2019-03-02

**Authors:** Luis Gabriel Rivera Calderón, Priscila Emiko Kobayashi, Rosemeri Oliveira Vasconcelos, Carlos Eduardo Fonseca-Alves, Renée Laufer-Amorim

**Affiliations:** 1 Department of Veterinary Pathology, School of Agricultural and Veterinarian Sciences, São Paulo State University (Unesp), Jaboticabal, São Paulo 14884-900, Brazil; griveramvz@gmail.com (L.G.R.C.); pri_kobayashi@hotmail.com (R.O.V.); 2 Department of Veterinary Clinic, School of Veterinary Medicine and Animal Science, São Paulo State University (Unesp), Botucatu, São Paulo 18618-681, Brazil; rosemeri.vasconcelos@unesp.br; 3 Department of Veterinary Surgery and Anesthesiology, School of Veterinary Medicine and Animal Science, São Paulo State University (Unesp), Botucatu, São Paulo 18618-681, Brazil; carlos.e.alves@unesp.br

**Keywords:** dog, prostatic tissue, extracellular matrix, picrosirius, immunohistochemistry

## Abstract

This study aimed to investigate collagen (Coll-I, III, IV) and elastin in canine normal prostate and prostate cancer (PC) using Picrosirius red (PSR) and Immunohistochemical (IHC) analysis. Eight normal prostates and 10 PC from formalin-fixed, paraffin-embedded samples were used. Collagen fibers area was analyzed with ImageJ software. The distribution of Coll-I and Coll-III was approximately 80% around prostatic ducts and acini, 15% among smooth muscle, and 5% surrounding blood vessels, in both normal prostate and PC. There was a higher median area of Coll-III in PC when compared to normal prostatic tissue (*p* = 0.001 for PSR and *p* = 0.05 for IHC). Immunostaining for Coll-IV was observed in the basal membrane of prostate acini, smooth muscle, blood vessels, and nerve fibers of normal and PC samples. Although there was no difference in Coll-IV area between normal tissue and PC, tumors with Gleason score 10 showed absence of Coll-IV, when compared to scores 6 and 8 (*p* = 0.0095). Elastic fibers were found in the septa dividing the lobules and around the prostatic acini of normal samples and were statistically higher in PC compared to normal tissue (*p* = 0.00229). Investigation of ECM components brings new information and should be correlated with prognosis in future studies.

## 1. Introduction

Cancer is the second leading cause of mortality worldwide. In men, prostate cancer (PC) is the third most common malignant neoplasia (after non-melanoma skin cancer and lung cancer) [1]. In the advanced stage, human PC often shows metastasis to bones and resistance to anti-androgen treatment [2]. Similar to the men, dogs spontaneously develop PC [3,4]. In dogs, PC it is a very aggressive and highly metastatic disease [5]. Usually, bone metastasis is diagnosed at a late stage of highly aggressive tumor subtypes [3]. Due to similarities in the clinical and pathologic aspects of PC in both species, some authors suggest that the dogs may be considered a good model for the study of human PC [4,6,7].

Recently, has been demonstrated in the human PC an interaction between the tumor cells and the proteins of extracellular matrix (ECM). The ECM fibers play an important role in PC development and progression [8,9]. The ECM is a complex network of macromolecules [10]. The major constituents of ECMs are proteoglycans and fibrous proteins (collagen, elastin, fibronectin and laminin) [11]. Collagen (Coll) is the most common protein of the ECM [10]. The main collagen function is to provide structure, support and tensile strength, as well as, regulation of cell adhesion, chemotaxis, migration and direct tissue development [12]. In some human cancers, such as breast, colon and prostate, occurs a formation of abundant collagenous stroma (reactive stroma) in their tumor microenvironment, responsible for the tumor metastatic process [8,13,14]. High density of type I collagen (Coll-I) and degradation of type IV collagen (Coll-IV) are frequently observed in solid cancer, associated with metastasis [9,15,16,17,18]. In veterinary medicine, few studies were conducted to understand the relationship between cancer and the collagens fibers compared to human medicine [19]. Due to the lack of information regarding tissue ECM in canine PC, this study aimed to characterize and compare the composition and distribution of collagen fibers and elastin in the normal prostate and canine PC, using PSR and immunohistochemical test.

## 2. Materials and Methods

### 2.1. The Subjects

Eight canine normal prostates and 10 PC were retrieved from the archives of the Veterinary Pathology Service, FMVZ, UNESP, Botucatu, SP, Brazil. The prostates were collected from necropsies from animals that had an interval between death and necropsy less than six hours. Formalin-fixed paraffin-embedded (FFPE) samples from canine prostatic tissue were sectioned for histological diagnosis, which was performed by three pathologists (LGRC, CEFA, PEK), at the same time, in a multi-head microscopy. The histopathological classification was performed according to the human WHO from Tumors of the Urinary System and Male Genital Organs [20], which was recently adapted to canine PC [4]. The Gleason score was established according to Palmieri and Grieco [6] (Table S1). This study was approved by the institutional committee for the use of animals in research (#10.162/2016).

PC samples (10/10) were from intact male dogs, with age raging 8 up to 12 years. Six out of 10 samples were from mixed breed dogs, two cases (2/10) were from a German Shepherd dog, one case (1/10) from a Brazilian Mastiff dog and the other one from a Poodle dog (1/10). Regarding the clinical signs, five out of 10 patients showed tenesmus, three dogs had lameness (3/10) and two dogs (2/10) had loss of appetite. Five out of 10 patients had metastasis at the diagnosis and all dogs (10/10) died due to PC.

Normal samples were collected during necropsy of dogs with no clinical signs of prostatic disease. All dogs (8/8) were intact male dogs with age ranging from 7 up to 11 years old. The same interval between death and necropsy was used for sample collection.

### 2.2. Picrosirius (PSR)

The slides were deparaffinized in xylene and rehydrated in alcohol. After, PSR staining was performed using a commercial kit (HistokitTM, Easypath, SP, Brazil), according to manufacturer’s instructions. The slides were examined in an optical microscopy with polarized light (Axio Imager A1, Zeiss^®^, Göttingen, Germany). The collagen fibers that presented red-orange birefringence were considered type I, while the collagen fibers with green birefringence were interpreted as type III (Coll-III) [21].

### 2.3. Immunohistochemistry (IHC)

The slides were subject to immunohistochemical using the peroxidase method. The antibodies, antigen retrieval, dilutions and incubation period are described in the Table 1. Endogenous peroxidase activity was inhibited with 4% hydrogen peroxide in methanol for 10 min at room temperature (RT). Then, the slides were treated with protein block serum-free for 15 min RT (Dako, Carpinteria, CA, USA). In each step, the slides were washed with Tris-buffered saline (pH 7.4). A LSAB system was used as secondary antibody; applied for 1 h at RT, according to manufacturer’s instructions (Dako, Carpinteria, CA, USA). Peroxidase activity was revealed with 3’,3’-Diaminobenzidinechromogen (DAB, Substrate System, Dako, Carpinteria, CA, USA). For the counterstained, Harris’s hematoxylin was used. As negative control, primary antibodies were replaced by Tris-buffered saline solution. A canine normal skin tissue was used as positive control tissue for all antibodies.

### 2.4. Interpretation of PSR and IHC Staining

In the PC samples, areas with a higher percentage of neoplastic cells and minimal density of inflammatory cells were selected. In the normal samples, samples were collected from the peripheral region of the prostate gland, avoiding areas close to the median septa, according to Ruetten et al. [22]. For normal and PC samples, it was captured five fields (20× magnification) with a digital camera (Axioncam MRc, Zeiss^®^ Vision, Göttingen,, Germany) for each protein and technique (IHC and PSR). The stained areas were analyzed with ImageJ 1.49v software (National Institutes of Health, available online: https://imagej.nih.gov/ij/index.html) and were assessed by setting a threshold, using the Image J threshold tool in accordance with the procedure described by Bauman et al. [23]. Briefly, the staining distribution and intensity of the collagens and elastic fibers were evaluated in both normal canine prostate and PC. For PSR staining, it was used a manual thresholding of hue (121–179), saturation (20–255), and brightness (10–255) values in ImageJ [23]. For each marker in immunohistochemistry, we established a threshold values as follow: Coll-I: hue (0–170), saturation (69–255), and brightness (90–181), Coll-III: hue (111–176), saturation (10–98), and brightness (37–157), Coll-IV: hue (65–255), saturation (90–178), and brightness (101–255) and elastin: hue (0–146), saturation (0–175), and brightness (0–209).

### 2.5. Data Analysis

Descriptive statistics were used to define the median and percentile of Coll-I, Coll-III and elastin in normal and canine PC. For statistical propose, we grouped samples with Gleason score 6 and 8 and compared with samples with Gleason score 10. Mann-Whitney U test was applied to compare the area percentage among normal and canine PC. Statistical significance was set at *p* ≤ 0.05. All statistical analysis was done using GraphPad Prism 8 (GraphPad Software Inc., La Jolla, CA, USA).

## 3. Results

**Figure 1:**
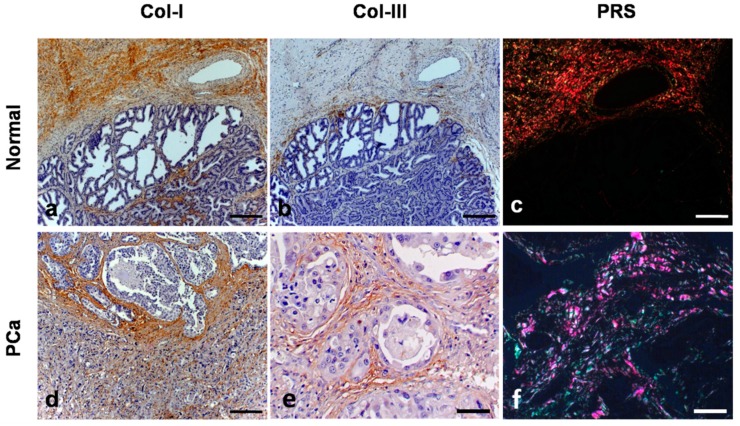
The immunohistochemistry and Picrosirius red (PSR) stain in normal tissue and canine PC. (**a**): immunostaining of Coll-I in the stroma of normal prostate (case No. 3). (**b**): immunostaining of Coll-III in the stroma of the normal prostatic tissue (case No. 3). (**c**): PSR staining observed in an optical microscopy with polarized light, the collagens fibers present red-orange birefringence (Coll-I) and green birefringence (Coll-III) in a smaller amount (case No. 3). (**d**): immunostaining of Coll-I in the stroma of prostatic neoplastic tissue (case No. 11). (**e**): immunostaining of Coll-III in the stroma of canine PC (case No. 11). (**f**): PSR staining in the canine PC with similar amounts of Coll-I and Coll-III (case No. 11).

Five out of 10 PC samples had Gleason 8 (5/10), four had Gleason score 10 (4/10) and one Gleason score 6 (1/10). The mean survival time was 152.1 days (±134.8). All prostatic samples (18/18) stained with PSR and the Coll-I was more abundant than Coll-III (Figure 1). The median expression of Coll-I by PSR in normal samples was 1.89 (1.196–3.839) and in PC samples was 2.24 (1.358–2.834). There was no statistical difference of Coll-I expression between normal and PC samples. Regarding the IHC for Coll-I, we also identified a higher proportion of Coll-I compared to Coll-III (Figure 1). The median expression of Coll-I in normal samples by IHC was 4.73 (1.367–8.414) and 6.18 (1.577–17.572) for PC samples. We identified a positive correlation of Col-I expression between PSR and IHC techniques (R = 0.6185; *p* = 0.05). Thus, although the results are numerically different for both techniques, there is a correlation of the results. Besides that, we evaluated the distribution of the Coll-I thought the normal prostate. Approximately 80% of the Coll-I was located surrounding prostatic ducts and acini, 15% among smooth muscle and 5% around blood vessels (Figure 1). We also did not find statistical difference of Coll-I immunoexpression between normal and PC samples. Comparing Coll-I expression between samples with Gleason score 10 and 8/6, we did not find statistical difference (*p* = 0.761).

Regarding Coll-III expression, we identified positive stain in all prostatic samples (18/18) for both techniques. PC samples showed a higher expression of Coll-III than normal samples by PSR (*p* = 0.001) and IHC (*p* = 0.05). The median expression for Coll-III expression in normal samples was 1.64 (0.975–3.329) and 2.25 (1.067–3.605) for PC samples, using PSR. We identified a strong positive correlation (R = 0.7805; *p* = 0.007) of Coll-III expression between PSR and IHC techniques. Thus, samples with high Coll-III by PSR technique also showed a higher Col-III expression by IHC. We also qualitatively assessed the Coll-III distribution among the prostatic tissues. In both normal and PC samples, 80% of the Coll-III was located surrounding prostatic ducts and acini, 15% among smooth muscle and 5% around blood vessels (Figure 1). Table 2 shows the median, 25% percentile and 75% percentile values of Coll-I and Coll-III according to the diagnosis group and test applied. There was no statistical difference regarding the Coll-III expression and the Gleason scores (*p* = 0.0654).

**Figure 2:**
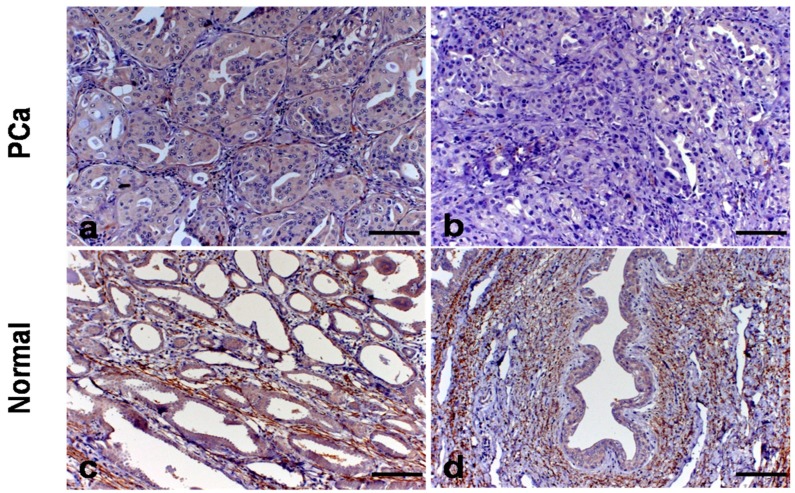
Immunostaining for Coll-IV and elastin in canine prostatic tissue. (**a**): Coll-IV immunostaining in the basal membrane of canine PC with cribiforme pattern (case No. 15). (**b**): Absence of Coll-IV immunostaining in canine PC with solid pattern (case No. 12). (**c**): Elastin fibers around the prostatic acini of normal samples (case No. 5). (**d**): High amount of elastin fibers around the urethra (case No. 5).

Immunostaining for Coll-IV was observed in the basal membrane (BM) of prostate acini, smooth muscle, blood vessels, and never fibers of normal and PC samples, although it was discontinuous in BM (Figure 2). It was observed a Coll-IV immunostaining surrounding continuously the blood vessels in both normal and PC samples. The distribution of Coll-IV was approximately 70% in the BM, 15% in smooth muscle, 10% in blood vessels BM and 5% in nerve fibers in both groups. When we compared the normal prostate with PC samples, we did not find statistical difference, concerning Coll-IV expression (*p* = 0.2135). However, it was identified absence of Coll-IV immunostaining in the tumors with Gleason score 10 compared to tumors with Gleason score 6 and 8 (*p* = 0.0095) (Figure 2). The results of Coll-IV expression are described in Table 3.

Immunostaining for elastin was observed with similar distribution than Coll-IV, around blood vessels in normal tissues and PC. Elastic fibers were found in the septa dividing the lobules and around the prostatic acini of normal samples. A high amount of elastic fibers was observed around the ducts and the urethra in normal and canine PC (Figure 2). Higher expression of Elastin in PC samples was identified compared to normal samples (*p* = 0.00229). There was no statistical difference comparing Gleason score 10 with Gleason 6 and 8 samples (*p* = 0.897).

## 4. Discussion

Changes in the cellular interactions, between the stroma and epithelial cells, play a crucial role in the carcinogenic process and progression of different human neoplasms such as prostate, breast and colon [24,25,26,27]. In cancer, the EMC is a network of macromolecules that allows the cellular evasion towards the defense of the organism, besides helping in their metastatic process [10]. Collagens fibers are important components of ECM remodeling the tumor microenvironment, and it is known that their degradation and redeposition promote tumor infiltration, angiogenesis, invasion and migration [11]. ECM pattern changes throughout tumor initiation and clinical disease, and it also influences cancer cell behavior and abilities to proliferate and metastasizes [28].

In this study, we identified and characterized collagen and elastin fibers in the normal prostate and canine PC (inside the tumor) using PSR and IHC tests. We also compared the correlation of both tests to identify Coll-I and III expression. Coll-I expression was not statistically different when compared normal prostates and canine PC samples using both techniques. Although PSR is less sensitive for staining of collagen fibers, the positive correlation between these two techniques, allows us to suggest that PSR is cheaper and routinely used in some histology laboratories, and can be a good choice. In humans, Bauman et al. [23] evaluated the Coll-I content in normal prostates and benign prostatic hyperplasia (PH) by PSR staining, with no statistical difference. We did not find studies evaluating collagens fibers with PSR and IHC simultaneously, in both human and canine PC. Regarding the immunostaining of Coll-I, statistical difference was also not identified comparing normal and PC samples. However, in PC samples, we identified a higher median of Coll-I expression. The lack of statistical difference can be related to the low number of cases and it also corroborates with Bauman et al. [23] results.

Wegner et al. [29] performed a study with PSR in the prostate gland of C57BL/6J mice. They found that fluorescent PSR imaging was more sensitive than polarized light for identifying the collagen fibers. In addition, Fluorescent PSR imaging was compatible with the collagen expression by IHC test. The fluorescent PSR imaging method seems to be promising, but it must still be studied in the comparative oncology. Fewer studies with FFPE samples of canine tumors were conducted to analyze the collagens fibers by PSR [19,30,31]. Bedoya et al. [32] used PSR staining in canine squamous cell carcinomas (SCCs), classified in well and poorly differentiated. The percentage of Col-I was approximately 30% for low- and high-grade SCCs. Their results showed a higher percentage of collagen fibers than observed in our study, comparing normal and canine PC, but these are different tumors with different patterns and locations.

The interaction between the EMC components and metastatic progression is widely studied in human cancers. However, the literature lack information regarding the collagen fibers and elastin expression in human and canine PC tissues. The previous human literature is focused on the role of EMC components in prostate cancer cells [33,34], instead of human prostatic tissue [35,36]. The Type I collagen degradation product (ICTP) was previously investigated in human PC, predicting bone metastasis [35]. However, ICPT expression is evaluated in serum and these authors did not evaluate collagen I expression in the prostatic samples. In this study, we evaluated the Coll-I immunoexpression in normal and neoplastic prostates, and did not study metastatic disease, which could be interesting information.

Coll-I and III gene and protein expression were previously evaluated in human PC, using RT-qPCR and IHC [37]. These authors also correlated Coll-I expression with Gleason score. No correlation was found between protein and gene expression for both collagens. However, the IHC analysis showed that Coll-I and Coll-III was significantly reduced in PC, in all Gleason scores, when compared to benign areas. In our work, we found higher Coll-I and Coll-III expression in PC compared to normal prostatic tissue by PSR and IHC tests, but only Col-III expression was significantly different. This is an opposite result compared to Duarte et al. [37].

One explanation for lower Coll-I and Coll-III found by Duarte et al. is that collagen reduction in PC could be the result of high metalloproteinase (MMP) activity [37]. MMPs have been correlated with tissue invasion and tumor progression of different cancers [38]. In human PC, tissue inhibitors of matrix metalloproteinases (TIMPs) and MMPs dysregulation are caused by a significant gain of MMP-2 and 9 expression and TIMP-1 loss [39]. The increased MMP-2 and 9 expression leads to the degradation of collagen fibers being the evaluation of Coll-I expression tricky. In canine PC, MMP-2 and MMP-9 were previously evaluated and the authors found overexpression when compared PC to normal prostate. However, the authors did not compare the results with collagen expression [40], as well as we did not measure MMPs in the present study. Based on this previous description in canine PC [39], MMP-2 and 9 overexpression can induce collagen degradation interfering in the collagen evaluation by IHC and PSR. EMC is generated in the microenvironment dynamically, according to physiological and pathological conditions (for example: age, hormone deprivation, inflammation, tumor progression and others). Our results represent a specific moment of the tumor, and not the dynamic protein expression in different tumor phases, such as growth, progression and invasion. Hypothetically, future biopsies could show higher MMP expression and lower Collagen expression.

Concerning Coll-I and III role in cancer, there is evidence that the stiffness of the microenvironment facilitates invasion and migration [41]. Epithelial cancer cells can migrate and invade basal membrane and surrounding stroma easily, in an environment rich with collagen fibers [42]. Also, higher collagen concentration can stimulate epithelial tumor cell to undergo epithelial-mesenchymal transition (EMT), corroborating with the malignant and metastatic potential [43]. So, it is not surprising that canine PC had higher Coll-I and III area than normal prostatic tissue, and we had 5/10 (50%) of the dogs with metastatic disease.

In a study measuring a Coll-I metabolite (N-terminal telopeptide-Tx) in serum from human patients with PC, this protein was correlated with tumor biochemical recurrence, stage of disease and bone metastasis [38]. Thus, we can assume that in a group of non-metastatic tumors, Coll-I might not have an important role, as in metastatic PC. We compared tumor with metastasis at the diagnosis (N = 5) versus non-metastatic tumors regarding all markers (data not shown). However, we did not find statistical difference between both groups, probably due to the low number of samples.

A previous study evaluated the difference in Coll-I expression “in vitro” in PC cells derived from bone marrow (bone metastasis PC cells) and from other metastatic tissues [41]. This study demonstrated a cellular reorganization (alterations in adhesion, elasticity and cytoskeletal organization) mediated by Coll-I and fibronectin [44]. The ECM expression is dynamic through the tumor life [45], so, maybe, when gaining metastatic abilities, Coll-I could be even higher than normal tissue, or our results could present the initiation of a metastatic phenotype.

Coll-IV was slighted decreased in canine PC, when compared to normal prostatic tissue, with no statistical difference. Since most of Coll-IV fibers was concentrated in the BM in both PC and normal tissue, the difference found is mainly related to this layer. It is expected to find reduced Coll-IV in the BM of neoplastic acinus, since this is a hallmark of carcinomas [27]. Coll-IV in the BM is not just a scaffold for the epithelial and endothelial cells, it also modulates cellular differentiation and proliferation [41]. Changes in Coll-IV can contribute to cancer progression, by leading to changes in cell polarity, proliferation, invasiveness and survival [46].

When BM, surrounding epithelial cancer cells, is lacking, it allows cancer cells to directly interact with stromal components, especially cancer-associated fibroblasts (CAFs), changing not only the tumor cell behavior, but also the cancer microenvironment [47]. Concerning Gleason grade and Coll-IV expression, tumors with higher Gleason grade (10) showed absence of Coll-IV compared to scores 6/8. Babichenko et al. [48] found that Coll-IV fibers disappeared with the increase of Gleason score. They also reported less Coll-IV immunoexpression in PC compared to PH.

The pattern of Col-IV expression in this study was similar to normal human prostate and PC [48]. An interesting finding was that solid canine PC had lower/absent Coll-IV expression than other PC patterns and normal tissues. Sinha et al. [49] found that Coll-IV immunostaining was less uniform or absent in the BM of poorly-differentiated human PC when compared to well-differentiated PC, HBP and normal human prostates. Similar results were also observed in feline and canine mammary tumors as well as in canine hemangiosarcoma [30,31]. In an experimental study of gerbil model of prostate carcinogenesis, the authors found proteolytic degradation of Coll-IV in a patchy pattern in the acini, indicating BM rupture [50].

We found a normal pattern [51] of elastic fibers in canine prostate (in the septa dividing the lobules, around the alveoli, ducts and the urethra). The interesting result was that canine PC, had a statistical higher median of elastin fibers (*p* = 0.00229) than normal tissue. Elastin fibers are involved in tumor invasion and metastasis [52], cell proliferation, adhesion, apoptosis and angiogenesis [53].

There is an important cross-talk between elastic fiber peptides and cells like fibroblast (CAFs), lymphocytes, macrophages, smooth muscle and endothelial cells (tumor microenvironment), acting as a pro-tumorigenic protein [54]. There are no reports of elastin fibers and prostatic cancer in different species, so the biological role of the increase of elastic fibers should be further investigated.

The collagen and elastin patterns in the prostate gland are dynamic and important differences can be found in different species. Besides that, the prostate gland anatomy, the animal breed, age and castration status can lead in different collagen and elastin fiber patterns [22]. In this study, we lack information regarding the region of the prostate gland (periurethral, peripheral or of the prostate biopsy) that the tissue specimens were collected. Thus, we strongly recommend the annotation of each region that the prostatic tissue was collected before evaluating the EMC components in canine prostate.

This study brings new information to veterinary literature, concerning some ECM components from canine PC. There are some points that could have been addressed, such as carcinoma surrounding fibrous tissue measurement, study of the metastasis and correlation with prognosis, so there is still a gap to be fulfilled.

## 5. Conclusions

PSR and IHC tests have a similar role in the evaluation of distribution and percentage area of collagen in normal and neoplastic canine prostate. There is a change in ECM profile in canine PC, with higher collagen III and elastin fiber expression in PC compared to normal canine tissue. Collagen IV higher expression correlates with higher Gleason scores in canine PC, so the investigation of ECM components brings new information and should be correlated with prognosis in future studies.

## Figures and Tables

**Table 1:** Primary antibodies, retrieval antigen, dilution and incubation period used in the immunohistochemistry (IHC) test.

Primary Antibody	Antigen Retrieval	Dilution	Incubation Period
Collagen I, rabbit, Novotec	Citrate buffer pH 6.0, microwave, twice for 5 min	1:1000	Overnight at 4 °C
Collagen III, rabbit, Novotec	Pepsin 2%, pH 1.4 in oven for 10 min at 60 °C after for 30 min at 37 °C.	1:1000	Overnight at 4 °C
Collagen IV, rabbit, Biorbyt	Pepsin 2%, pH 1.4, in oven for 10 min at 60 °C after for 30 min at 37 °C.	1:1000	Overnight at 4 °C
Elastin (BA-4), mouse, Santa Cruz.	Citrate buffer pH 6.0, pressure cooker (Pascal^®^, Dako, Carpinteria, CA, USA)	1:100	Overnight at 4 °C

**Table 2:** Median, 25% percentile and 75% percentile values of area percentage staining for collagen (Coll-I) and Coll-I in the normal and canine prostate cancer (PC), according to the method used.

		PSR Test				IHC Test			
		25%	Median	75%	*p*	25%	Median	75%	*p*
Coll-I	N	1.25	1.89	2.27	0.1298	2.85	4.73	8.03	0.3159
	PC	2.09	2.24	2.43		3.31	6.18	8.56	
Coll-III	N	1.33	1.64	2.06	0.001	1.81	3.22	5.03	0.05
	PC	1.68	2.25	3.11		3.72	5.07	6.44	

Coll-I: Collagen I, Coll-III: Collagen III, N: Normal, PC: Prostate cancer.

**Table 3:** Median, 25% percentile and 75% percentile values of area percentage staining for Coll-IV and elastin in the normal prostate and canine PC samples.

	Group	IHC Test			
		25%	Median	75%	*p*
Coll-IV	Normal	1.11	1.41	1.72	0.2135
	PC	0.58	1.14	1.61	
Elastin	Normal	0.25	0.26	0.42	0.00229
	PC	0.28	0.43	0.51	

## Supplementary Materials

The following are available online at https://www.mdpi.com/2306-7381/6/1/22/s1, Table S1: Histological type of 10 canine prostatic carcinomas, according to Eble et al. 2004 and Palmieri et al. 2014.

## References

[1] Ferlay J., Soerjomataram I., Ervik M., Dikshit R., Eser S., Mathers C., Rebelo M., Parkin D.M., Forman D., Bray F. (2014). Cancer incidence and mortality worldwide: Source, methods and major patterns in GLOBOCAN 2012. Int. J. Cancer.

[2] Mundy G. (2002). Metastasis to bone: Causes, consequences and therapeutic opportunities. Nat. Rev. Cancer.

[3] Leroy B.R., Northrup N. (2009). Prostate cancer in dogs: Comparative and clinical aspects. Vet. J..

[4] Palmieri C., Lean F.Z., Akter S.H., Romussi S., Grieco V. (2014). A retrospective analysis of 111 canine prostatic samples: Histopathological findings and classification. Res. Vet. Sci..

[5] Argyle D.J. (2009). Prostate cancer in dogs and men: Unique opportunity to study the disease. Vet. J..

[6] Palmieri C., Grieco V. (2015). Proposal of Gleason-like grading system of canine prostate carcinoma in veterinary pathology practice. Res. Vet. Sci..

[7] Fonseca-Alves C.E., Kobayashi P.E., Rivera Calderón L.G., Felisbino S.L., Rinaldi J.C., Drigo S.A., Rogatto S.R., Laufer-Amorim R. (2018). Immunohistochemical panel to characterize canine prostate carcinomas according to aberrant p63 expression. PLoS ONE.

[8] Palumbo A., Ferreira L.B., de Souza P.A.R., Oliveira F.L., Pontes B., Viana N.B., Machado D.E., Palmero C.Y., Alves L.M., Gimba E.R. (2012). Extracellular matrix secreted by reactive stroma is a main inducer of pro-tumorigenic features on LNCaP prostate cancer cells. Cancer Lett..

[9] Penet M.F., Kakkad S., Pathak A.P., Krishnamachary B., Mironchik Y., Raman V., Solaiyappan M., Bhujwalla Z.M. (2016). Structure and function of prostate cancer dissemination-permissive extracellular matrix. Clin. Cancer Res..

[10] Frantz C., Stewart K.M., Weaver V.M. (2010). The extracellular matrix at a glance. J. Cell. Sci..

[11] Theocharis A.D., Skandalis S.S., Gialeli C., Karamanos N.K. (2016). Extracellular matrix structure. Adv. Drug Deliv. Rev..

[12] Rozario T., DeSimone D.W. (2010). The extracellular matrix in development and morphogenesis: A dynamic view. Dev. Biol..

[13] Tuxhorn J.A., Ayala G.E., Rowley D.R. (2001). Reactive stroma prostate cancer progression. J. Urol..

[14] Zhang K., Corsa C.A., Ponik S.M., Prior J.L., Piwnica-Worms D., Eliceiri K.W., Keely P.J., Longmore G.D. (2013). The collagen receptor discoidin domain receptor 2 stabilizes SNAIL1 to facilitate breast cancer metastasis. Nat. Cell Biol..

[15] Zeng Z.S., Cohen A.M., Guillem J.G. (1999). Loss of basement membrane type IV collagen is associated with increased expression of metalloproteinases 2 and 9 during human colorectal tumorigenesis. Carcinogenesis.

[16] Tanjore H., Kalluri R. (2006). The role of type IV collagen and basement membranes in cancer progression and metastasis. Am. J. Pathol..

[17] Provenzano P.P., Inman D.R., Eliceiri K.W., Knittel J.G., Yan L., Rueden C.T., White J.G., Keely P.J. (2008). Collagen density promotes mammary initiation and progression. BMC Med..

[18] Li A., Zhou T., Guo L., Si J. (2010). Collagen type I regulates ß-catenin tyrosine phosphorylation and nuclear translocation to promote migration and proliferation of gastric carcinoma cells. Oncol. Rep..

[19] Case A., Brisson B.K., Durham A.C., Rosen S., Monslow J., Buza E., Salah P., Gillem J., Ruthel G., Veluvolu S. (2017). Identification of prognostic collagen signatures and potential therapeutic stromal targets in canine mammary gland carcinoma. PLoS ONE.

[20] Eble J.N., Sauter G., Epstein I.A. (2004). Sesterhenn Pathology and Genetics of Tumours of the Urinary System and Male Genital Organs.

[21] Coleman R. (2011). Picrosirius red staining revisited. Acta Histochem..

[22] Ruetten H., Wegner K.A., Romero M.F., Wood M.W., Marker P.C., Strand D., Colopy S.A., Vezina C.M. (2018). Prostatic collagen architecture in neutered and intact canines. Prostate.

[23] Bauman T.M., Nicholson T.M., Abler L.L., Eliceiri K.W., Huang W., Vezina C.M., Ricke W.A. (2014). Characterization of fibrillar collagens and extracellular matrix of glandular benign prostatic hyperplasia nodules. PLoS ONE.

[24] Yang F., Tuxhorn J.A., Ressler S.J., McAlhany S.J., Dang T.D., Rowley D.R. (2005). Stromal expression of connective tissue growth factor promotes angiogenesis and prostate cancer tumorigenesis. Cancer Res..

[25] Martin M., Pujuguet P., Martin F. (1996). Role of stromal myofibroblasts infiltrating colon cancer in tumor invasion. Pathol. Res. Pract..

[26] Tuxhorn J.A., McAlhany S.J., Dang T.D., Ayala G.E., Rowley D.R. (2002). Stromal cells promote angiogenesis and growth of human prostate tumors in a differential reactive stroma (DRS) xenograft model. Cancer Res..

[27] Valastyan S., Weinberg R.A. (2011). Tumor metastasis: Molecular insights and evolving paradigms. Cell.

[28] Jones C.E., Hammer A.M., Cho Y., Sizemore G.M., Cukierman E., Yee L.D., Ghadiali S.N., Ostrowski M.C., Leight J.L. (2019). Stromal PTEN Regulates Extracellular Matrix Organization in the Mammary Gland. Neoplasia.

[29] Wegner K.A., Keikhosravi A., Eliceiri K.W., Venzina C.M. (2017). Fluorescence of picrosirius red multiplexed with immunohistochemistry for the quantitative assessment of collagen in tissue sections. J. Histochem. Cytochem..

[30] Benazzi C., Sarli G., Galeotti M., Marcato P.S. (1993). Basement membrane components in mammary tumours of the dog and cat. J. Comp. Pathol..

[31] Murakami M., Sakai H., Kodama A., Yanai T., Mori T., Maruo K., Masegi T. (2009). Activation of matrix metalloproteinase (MMP-2) by membrane type 1-MMP and abnormal immunolocalization of the basement membrane components laminin and type IV collagen in canine spontaneous hemangiosarcomas. Histol. Histopathol..

[32] Bedoya S.A.O., Conceição L.G., Viloria M.I.V., Loures F.H., Valente F.L., Amorim R.L., Silva F.F. (2016). Caracterização de colágenos tipos I e III no estroma do carcinoma de células escamosas cutâneo em cães. Arquivo Brasileiro de Medicina Veterinária e Zootecnia.

[33] Koutsilieris M., Sourla A., Pelletier G., Doillon C.J. (1994). Three-dimensional type I collagen gel system for the study of osteoblastic metastases produced by metastatic prostate cancer. J. Bone Miner. Res..

[34] Hall C.L., Dai J., van Golen K.L., Keller E.T., Long M.W. (2006). Type I collagen receptor (alpha 2 beta 1) signaling promotes the growth of human prostate cancer cells within the bone. Cancer Res..

[35] Kylmälä T., Tammela T.L., Risteli L., Risteli J., Kontturi M., Elomaa I. (1995). Type I collagen degradation product (ICTP) gives information about the nature of bone metastases and has prognostic value in prostate cancer. Br. J. Cancer.

[36] Burns-Cox N., Avery N.C., Gingell J.C., Bailey A.J. (2001). Changes in collagen metabolism in prostate cancer: A host response that may alter progression. J. Urol..

[37] Duarte A.H., Colli S., Alves-Pereira J.L., Martins M.P., Sampaio F.J.B., Ramos C.F. (2012). Collagen I and III metalloproteinase gene and protein expression in prostate cancer in relation to Gleason score. Int. Braz. J. Urol..

[38] Jablonka F., Alves Bda C., de Oliveira C.G., Wroclawski M.L., Szwarc M., de Oliveira Vitória W., Fonseca F., Del Giglio A. (2014). Serum crosslinked-*N*-terminal telopeptide of type I collagen (NTx) has prognostic implications for patients with initial prostate carcinoma (PCa): A pilot study. Clin. Chim. Acta.

[39] Brehmer B., Biesterfeld S., Jakse G. (2003). Expression of matrix metalloproteinases (MMP-2 and -9) and their inhibitors (TIMP-1 and -2) in prostate cancer tissue. Prostate Cancer Prostatic Dis..

[40] Faleiro M.B., Croce G.B., Toledo D.C., Rodrigues M.M., Batista A.C., Damasceno A.D., Brito L.A., Laufer-Amorim R., de Moura V.M. (2013). Matrix metalloproteinases 2 and 9 expression in canine normal prostate and with proliferative disorders. Ciência Rural.

[41] Timpl R. (1996). Macromolecular organization of basement membranes. Curr. Opin. Cell Biol..

[42] Cassereau L., Miroshnikova Y.A., Ou G., Lakins J., Weaver V.M. (2015). A 3D tension bioreactor platform to study the interplay between ECM stiffness and tumor phenotype. J. Biotechnol..

[43] Haage A., Schneider I.C. (2014). Cellular contractility and extracellular matrix stiffness regulate matrix metalloproteinase activity in pancreatic cancer cells. FASEB J..

[44] Docheva D., Padula D., Schieker M., Clausen-Schaumann H. (2010). Effect of collagen I and fibronectin on the adhesion, elasticity and cytoskeletal organization of prostate cancer cells. Biochem. Biophys. Res. Commun..

[45] Kai F., Laklai H., Weaver V.M. (2016). Force matters: Biomechanical regulation of cell Invasion and migration in disease. Trends Cell Biol..

[46] Bissell M.J., Hines W.C. (2011). Why don’t we get more cancer? A proposed role of the microenvironment in restraining cancer progression. Nat. Med..

[47] Miyazaki K., Oyanagi J., Hoshino D., Togo S., Kumagai H., Miyagi Y. (2019). Cancer cell migration on elongate protrusions of fibroblasts in collagen matrix. Sci. Rep..

[48] Babichenko I.I., Andriukhin M.I., Pulbere S., Loktev A. (2014). Immunohistochemical expression of matrix metalloproteinase-9 and inhibitor of matrix metalloproteinase-1 in prostate adenocarcinoma. Int. J. Clin. Exp. Pathol..

[49] Sinha A.A., Gleason D.F., DeLeon O.F., Wilson M.J., Limas C., Reddy P.K., Furcht L.T. (1991). Localization of type IV collagen in the basement membranes of human prostate and lymph nodes by immunoperoxidase and immunoalkaline phospatase. Prostate.

[50] Gonçalves B.F., Campos S.G., Costa C.F., Scarano W.R., Góes R.M., Taboga S.R. (2015). Key participants of the tumor microenvironment of the prostate: An approach of the structural dynamic of cellular elements and extracellular matrix components during epithelial-stromal transition. Acta Histochem..

[51] Marettová E. (2017). Immunohistochemical localization of elastic system fibres in the canine prostate. Folia Veterinaria.

[52] Krušlin B., Ulamec M., Tomas D. (2015). Prostate cancer stroma: An important factor in cancer growth and progression. Bosn. J. Basic Med. Sci..

[53] Scandolera A., Odoul L., Salesse S., Guillot A., Blaise S., Kawecki C., Maurice P., El Btaouri H., Romier-Crouzet B., Martiny L. (2016). The Elastin Receptor Complex: A Unique Matricellular Receptor with High Anti-tumoral Potential. Front. Pharmacol..

[54] Fülöp T., Larbi A. (2002). Putative role of 67 kDa elastin-laminin receptor in tumor invasion. Semin. Cancer Biol..

