# Supplementary material for: Characterization of Collagen Fibers (I, III, IV) and Elastin of Normal and Neoplastic Canine Prostatic Tissues
Source: Vet Sci. 2019 Mar 2;6(1):22. doi: 10.3390/vetsci6010022 (PMC6466295; doi:10.3390/vetsci6010022)
Supplement: Supplementary file 1 [file vetsci-06-00022-s001.pdf]

Table S2. Histological type of 10 canine prostatic carcinomas, according to Eble et al. 2004 and Palmieri et al. 2014.

Histologic Type	Case Number
**Single histological pattern**	
Cribriform with comedonecrosis	14, 16
Solid	12, 18
Small acinar/ductual	9, 10
**Mixed histological patterns**	
Solid, cribriform with comedonecrosis	11, 17
Cribriform with comedonecrosis, solid	13
Cribriform with comedonecrosis, cribriform without comedonecrosis	15
